# EFFECTS OF ADAPTIVE COMPUTER-BASED COGNITIVE TRAINING ON COMMUNITY-DWELLING ELDERS

**DOI:** 10.1093/geroni/igz038.918

**Published:** 2019-11-08

**Authors:** Guifang Guo, Huijuan Gongzhi

**Affiliations:** 1 School of Nursing, Peking University, Beijing, Beijing, China; 2 Qingdao Municipal Hospital, Qingdao, Shandong, China (People’s Republic)

## Abstract

This quasi-experimental designed study analyzed the effects of adaptive computer-based cognitive training among community-dwelling older adults. A 6-week (5 times/week) program was implemented with an intervention group (Difficulty Adaptive Training) and control group (Difficulty Fixed Training). General cognitive, memory, executive and attention functions were evaluated before (T1), completion (T2), and one month after intervention (T3). Sixty-one participants completed data collection. (1) General cognitive function: improved in both groups at T2, and T3, intervention group had better effect; (2) Memory function: improved in both groups in immediate, short and long-delayed recalls at T2 and T3, and recognition at T2. (3) Executive function: improved in both groups. Time of simple information processing was shortened at T2 and T3 in intervention group, at T3 in control group; TMT response inhibition was shortened at T2 and T3 in both groups. (4) Attention function: digit span forward was improved at T2 in intervention group.

